# The prevalence of multimorbidity with mental and physical health for people who experience homelessness: a systematic review

**DOI:** 10.1093/eurpub/ckaf144

**Published:** 2025-08-28

**Authors:** Natasha Chilman, Peter Schofield, Dionne Laporte, Amy Ronaldson, Jayati Das-Munshi

**Affiliations:** Department of Psychological Medicine, Institute of Psychiatry, Psychology & Neuroscience, King’s College London, London, United Kingdom; Faculty of Life Sciences and Medicine, King’s College London, London, United Kingdom; Centre for Society and Mental Health, King’s College London, London, United Kingdom; Population Health Improvement, UK (PHI-UK); Faculty of Life Sciences and Medicine, King’s College London, London, United Kingdom; Population Health Improvement, UK (PHI-UK); Department of Psychological Medicine, Institute of Psychiatry, Psychology & Neuroscience, King’s College London, London, United Kingdom; Centre for Society and Mental Health, King’s College London, London, United Kingdom; Population Health Improvement, UK (PHI-UK); Department of Health Service and Population Research, Institute of Psychiatry, Psychology & Neuroscience, King’s College London, London, United Kingdom; Department of Psychological Medicine, Institute of Psychiatry, Psychology & Neuroscience, King’s College London, London, United Kingdom; Centre for Society and Mental Health, King’s College London, London, United Kingdom; Population Health Improvement, UK (PHI-UK); South London and Maudsley NHS Foundation Trust, London, United Kingdom

## Abstract

Multimorbidity refers to the co-occurrence of multiple health conditions in a single individual. The objective of this systematic review was to synthesize and evaluate research on the prevalence of multimorbidity (including both mental and physical health conditions) for people who have experienced homelessness. MEDLINE, EMBASE, PsycINFO, Web of Science, and OpenGrey were searched for relevant studies between 1997 and 2025. Studies were included if the sample consisted of adults in high-income countries, where the exposure was current or former homelessness, and the outcome was multimorbidity including both mental and physical conditions. Random-effects meta-analyses were used to calculate pooled prevalence estimates. The studies were narratively synthesized, and quality assessed. The search retrieved 6043 papers, 30 of which were eligible for inclusion in the review. Most studies recruited participants from specialist homelessness services (n = 21). More than half of the study samples were over 75% male (N = 16). When excluding studies which applied non-probability sampling strategies, the pooled prevalence was 45% (95% CI, 25–66) for multimorbidity. There was a 34% (95% CI, 22–48) pooled prevalence for trimorbidity (co-occurring mental, physical, and substance/alcohol use conditions). High heterogeneity was observed across studies (*I*^2^ > 99%). To conclude, multimorbidity is highly prevalent for people who experience homelessness. There is a lack of research on multimorbidity for women who are or have been homeless, and for those who are not accessing specialist homelessness services. These findings demonstrate the need for the integration, collaboration, and co-ordination between services to support the multimorbid health needs of people who experience homelessness.

## Introduction

People who experience homelessness are at the sharpest end of health and social inequalities. The health profiles of people who experience homelessness have often been described as ‘complex’ and characterized by multimorbidity [1]. Multimorbidity is commonly defined as the presence of co-occurring diseases or conditions in a single individual [2]. The most complex form of multimorbidity is trimorbidity, defined by the co-existence of mental, physical and substance use conditions, which was coined to describe the health needs of people who are chronically homeless [3].

Multimorbidity is associated with several adverse outcomes for patients—including decreases in quality of life and wellbeing, and increases in disability and premature mortality [4]—and a substantially high economic burden on both the health system and wider society [5]. There is evidence that people with concurrent mental and physical morbidities have worse health-related quality of life and clinical outcomes, as well as an increased risk of premature mortality, compared to those with physical or mental morbidities only [4]. Furthermore, a lack of parity between mental and physical health in policy and practice [6], and stigma associated with mental health conditions [7], could add an additional dimension to experiences of multiple health conditions. The prevalence of mental health conditions in homeless populations is particularly high, at an estimated 76.2% (95% CI, 64.0–86.6) [8]. It has been argued that multimorbidity including both mental and physical conditions deserves special attention [9], and mental health conditions should at least be included in measures of multimorbidity [4, 10].

Previous systematic reviews have collated evidence on mental and physical health conditions separately from one another in homeless populations [1, 8, 11, 12]. There is a need to bring the totality of evidence on mental and physical multimorbidity and homelessness together to better understand the prevalence of multimorbidity for this group and to support the provision of integrated services. This systematic review aimed to ascertain the prevalence of multimorbidity (including both mental and physical conditions) for people who have experienced homelessness.

## Methods

The systematic review was prospectively registered on Prospero (ref CRD42021247591) and was designed to apply to multiple groups who are known to experience social exclusion [1], which was then refined to focus on homeless populations only.

### Search strategy

The following databases were searched for references published from 1st January 1997: MEDLINE, EMBASE, PsycINFO, Web of Science. Grey literature was also searched using OpenGrey. Date restrictions coincide with the establishment of the Social Exclusion Unit in the United Kingdom. Search terms were determined based on previous literature reviews [1, 13, 14] and common terminology for multimorbidity and homelessness. The search was restricted to studies published in English. The full search strategy is included in Supplementary File S1.

The first database search was conducted in April 2021, updated in September 2023 and January 2025. The updated searches were re-run on all databases except for OpenGrey, as this was decommissioned and no longer updated in September 2023 onwards.

Corresponding authors of included studies were contacted in September 2023 and provided with a list of included studies. They were asked to identify any additional reports for inclusion in the review. Backward and forward citation searches of papers in the review were conducted to identify further studies.

### Selection criteria

Quantitative case-control, cross-sectional, retrospective, and prospective study designs were eligible for inclusion.

Studies were included if: (1) the sample consisted of adults (>50% of the sample aged ≥18) in high-income countries; (2) the exposure was current or former homelessness; and (3) the outcome included the prevalence of multimorbidity, defined as multiple co-occurring mental and physical conditions. Studies were excluded where they assessed for physical or mental multimorbidity only, and where studies used weighted measures of multimorbidity. Studies were included where they assessed trimorbidity. Studies did not need to have a comparison group to be included; where studies did, this was defined in terms of the exposure (no experience of homelessness).

### Data extraction and synthesis

Duplicate records were removed. The titles and abstracts from a randomly selected 5% of the papers retrieved by the first search were double screened by N.C. and D.L. A Cohen’s Kappa statistic was calculated to assess for agreement. Discussions were held to resolve any papers where there were disagreements. Where disagreements could not be resolved, a third researcher (J.D.M.) reviewed the abstract to reach a majority decision. N.C. screened the remaining titles/abstracts. In the second phase of screening, full-text articles were then accessed and screened for eligibility by N.C. Study authors were contacted where additional information was needed.

Where the article met the inclusion criteria for the review, data extraction was carried out by N.C. using a standardized pro forma [15–17]. Where multiple reports stemmed from the same primary sample, these were counted as one study and described together during data extraction. Following quality guidance [18], data were double-extracted for 50% of included papers (by A.R.) to check for completeness and accuracy. Data extraction forms were cross-checked for agreement, and discussions were held to resolve any disagreements.

Studies were quality assessed using Kmet’s risk of bias assessment tool for quantitative studies [19]. The Kmet includes 14 quality criteria items, such as whether the research question is sufficiently described, and whether the analytic methods described are justified and appropriate. Quality assessments were completed by N.C., with the exception of one paper where N.C. was the lead author [20], which was quality assessed by D.L.

A proportional meta-analysis was conducted to generate summary estimates of multimorbidity prevalence and its variance [21] using the *metaprop* command in Stata 17 [22]. Due to anticipated differences between and within studies, a random effects model was used. To prevent estimates which were close to one from being excluded from the meta-analyses, a Freeman-Tukey transformation was applied to stabilize the variance. Heterogeneity was explored in all models using the *I*^2^ statistic, inspections of the forest plots, and stratified analyses. A funnel plot was not used to assess for publication bias as there is a lack of consensus on how these should be interpreted for proportion statistics [21] and previous research indicates that these models lack accuracy and interpretation guidance when used in proportional meta-analyses [23]. Authors were contacted regarding any unpublished works, which aimed to reduce publication bias to some extent.

## Results

**Figure 1.**
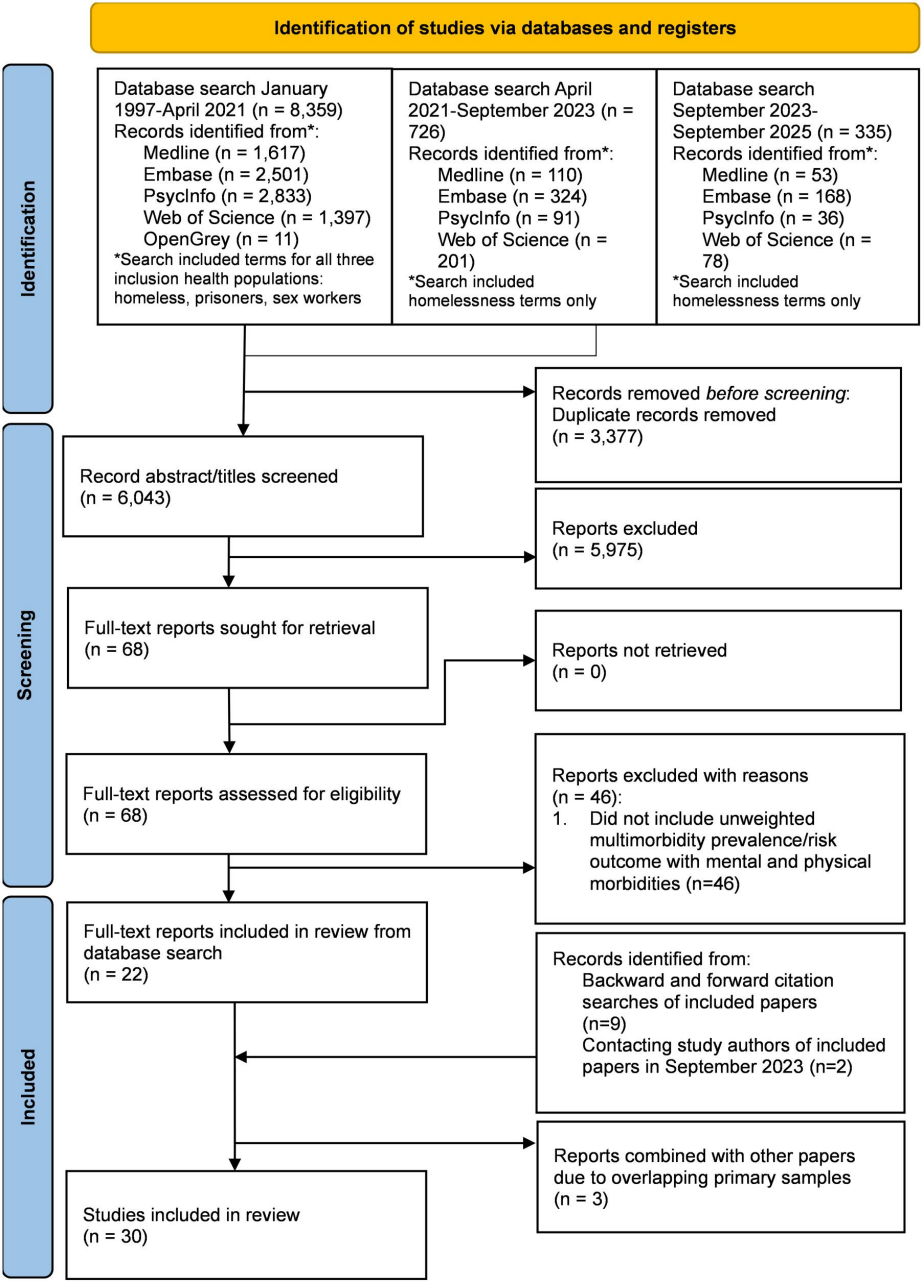
Preferred Reporting Items for Systematic Reviews and Meta-Analyses (PRISMA) flow diagram of the studies included at each stage of screening.

Figure 1 shows the PRISMA flow diagram. After de-duplication, 6043 titles and abstracts were screened. During double screening of 270 titles and abstracts, the two researchers reached a Cohen’s Kappa agreement of 0.66, indicating substantial agreement. Sixty-eight papers were eligible for full-text screening. In total, 46 of these full-text papers were excluded because they did not include an unweighted multimorbidity outcome including both mental and physical health conditions. Backward and forward citation searches of included papers identified nine further additional papers. After contacting study authors, two further papers were identified and eligible. This led to a total of 33 papers. Six papers were combined into three studies as they used the same datasets. In total, 30 studies were eligible for inclusion. Out of the 50% of studies which were double extracted, there was a high level of agreement (92.65%).

### Characteristics of included studies

Table 1 provides an overview of the characteristics of the included studies; Supplementary File S2 provides details on each included study. Studies were published between 2009 and 2024. Most studies were from the United States (*n* = 13), followed by the United Kingdom (*n* = 9). More than half of the included homeless samples were over 75% male (*n* = 16).

**Table 1.** Overview of included studies.^a^

	No. of studies (total N = 30)	% of studies
**Country**		
** United Kingdom**	9	30%
** United States**	13	43%
** Australia**	2	7%
** Canada**	2	7%
** Germany**	1	3%
** Hungary**	1	3%
** Netherlands**	1	3%
** Ireland**	1	3%
**Setting**		
** Specialist homeless service**	21	70%
** Other^b^**	9	30%
**Comparison group**		
** Non-homeless comparison**	6	20%
** No controls**	24	80%
**Sampling strategy**		
** Probability**	3	10%
** Non-probability**	13	43%
** Whole sampling frame**	14	47%
**Multimorbidity outcome**		
** ≥2 conditions**	12	40%
** Trimorbidity**	6	20%
** Continuous condition count**	4	13%
** Combination of above outcomes**	9	30%
**Experience of homelessness^c^**		
** Currently homeless**	26	86%
** Formerly homeless**	3	10%
** Combination of above**	1	3%
**Sample size^c^**		
** ≤50**	3	10%
** 51–250**	9	30%
** 251–1000**	9	30%
** 1000–15 920**	7	23%
** Not available/reported**	2	7%
**Sex^c^**		
** ≥75% male**	16	53%
** 50–74% male**	12	40%
** <50% male**	1	3%
** Not collected**	1	3%
**Age^c^**		
** Majority/mean < 50**	18	60%
** Majority/mean ≥ 50**	11	37%
** Not collected**	1	3%

a All studies were cross-sectional.

b Other settings include veteran nursing homes and services, mainstream healthcare services, humanitarian healthcare clinics, private household surveys, and people rough sleeping on the street.

c For the sample with experience of homelessness only (not including comparison group).

All but four studies assessed multimorbidity in populations who were currently experiencing homelessness, with the exceptions assessing multimorbidity either in people who were formerly homeless [20, 24, 25] or in both formerly and currently homeless people [26]. Most studies recruited or included samples from specialist homelessness services (*n* = 21). These included people accessing specialist primary care services [26–35], specialist secondary care, acute, and inpatient services [34, 36–38], hostels, hotels, shelters, or temporary accommodation [32, 39–45], outreach services [41, 46, 47], food halls [27], and supported housing [24, 48]. Some studies sampled from a combination of these settings [32, 45]. Other studies sampled from veterans’ nursing homes [25], veterans’ primary care services [26], mainstream health services [49–51], private households [20], and people sleeping on the street [43, 47, 52–54].

Studies were largely descriptive and lacked population controls (*n* = 24). Recruitment and sampling strategies varied across studies. Almost half of the included studies applied non-probability sampling methods, such as convenience and snowball sampling (*n* = 13). A similar number of studies included or accessed data for the whole sampling frame (*n* = 14), which meant that data were collected for all people in the researcher’s intended population (e.g. all people accessing a specialist homeless service). Most of these latter studies accessed data from electronic health records; with the exception of one study, where researchers spoke to service staff to collect data on all people accessing a service [44]. A small number of studies applied probability sampling strategies [20, 26, 52] (*n* = 3).

### Multimorbidity

Most studies defined multimorbidity as the presence of ≥2 health conditions (which could be mental, physical, or substance use conditions) in a single individual [20, 27–31, 33, 35, 38, 42, 43, 46–48, 50, 51, 54]. While some studies looked at the prevalence or risk of any multimorbidity according to this definition, several studies assessed for, trimorbidity (co-occurring mental health, physical health, and substance use conditions) [20, 24–26, 29, 35–38, 41, 44, 53] and, to a lesser extent, mental-physical multimorbidity (co-occurring mental *and* physical health conditions) [20, 28, 35–38]. Several studies calculated the average number of conditions per individual as a multimorbidity outcome [27, 29, 34, 35, 39, 40, 43, 49].

While several studies included any health conditions experienced by the sample [24, 32, 33, 36, 37, 43, 45–47, 51, 53], many studies focused on a pre-specified list of morbidities [20, 25–31, 34, 35, 38–42, 48–50, 52, 54]. The number of conditions included in pre-specified lists ranged from 10 to 41 conditions. Some of these studies used existing multimorbidity index lists, including the unweighted Elixhauser comorbidity index [55] and a list of conditions used in a study of primary care and multimorbidity in the general population [56]. However, many of these morbidity lists were determined by the investigators of each study, rather than established indexes. A total of 13 studies ascertained morbidities through sources and measures such as health records and validated measurement tools [24, 25, 28–31, 33–36, 38–40, 47, 49–51], 8 studies relied on self-reported conditions [30, 41, 42, 46, 48, 52–54], 2 studies ascertained morbidities from professionals’ reports [37, 44], and the remainder used a combination of these methods [20, 26, 27, 32, 43, 45].

### Risk of bias assessments

The Kmet quality assessment summary scores for each report are included in the Supplementary Materials. Kmet scores range from 0 to 1, with scores closer to 1 indicating higher quality and a lower risk of bias. When grouped by sampling strategy, the average score for studies which applied probability sampling methods, or those which accessed data for the whole sampling frame was 0.74 (SD = 0.14). Studies which applied non-probability sampling strategies generally scored slightly lower (average score = 0.62, SD = 0.09). These non-probability sampling studies were subject to sampling biases. Consequently, the following meta-analyses includes studies which applied probability sampling or included the whole sampling frame. Meta-analyses including studies which applied non-probability sampling studies are included in the Supplementary Materials.

### Meta-analyses

**Figure 2.**
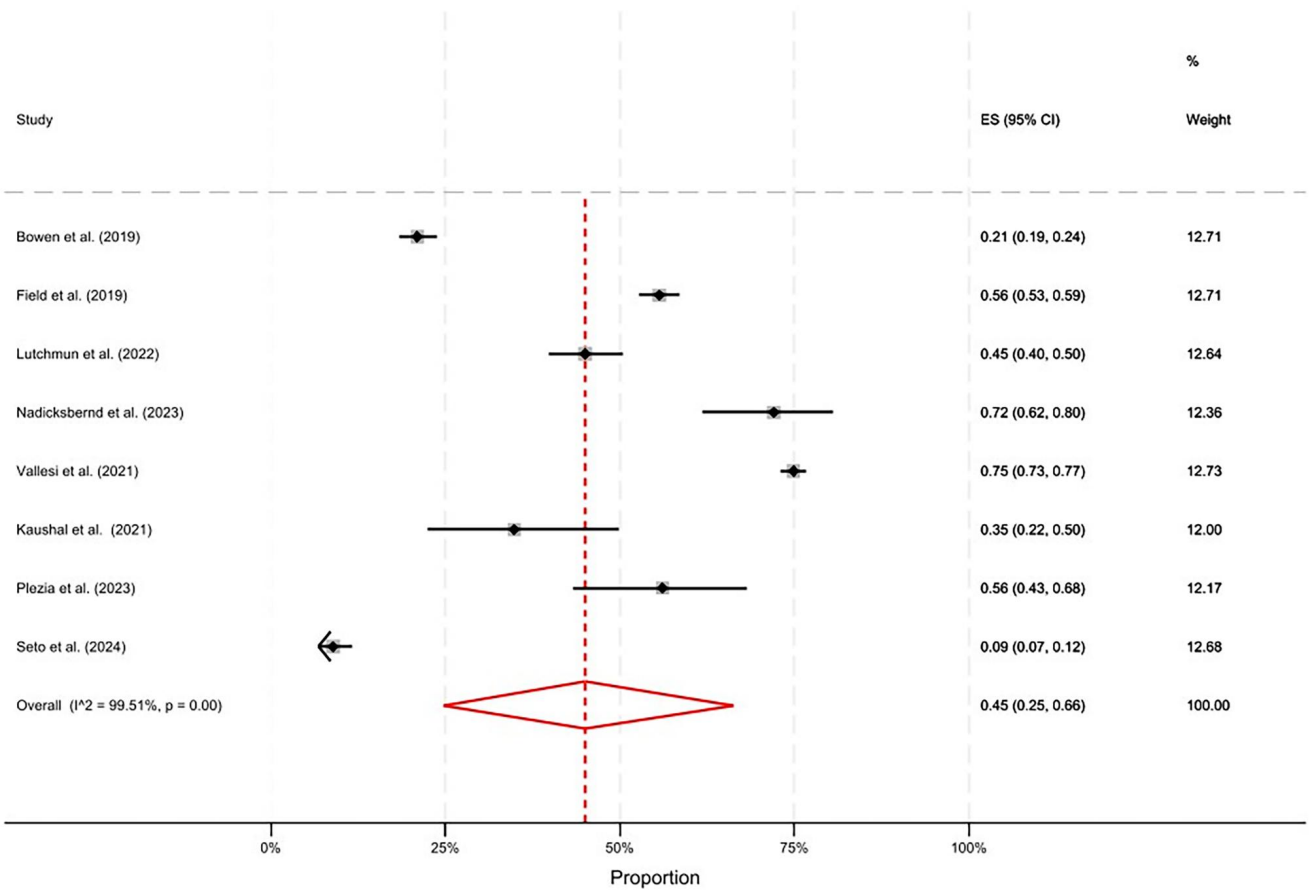
The pooled prevalence of multimorbidity for people with experience of homelessness, excluding non-probability sampling studies.

**Figure 3.**
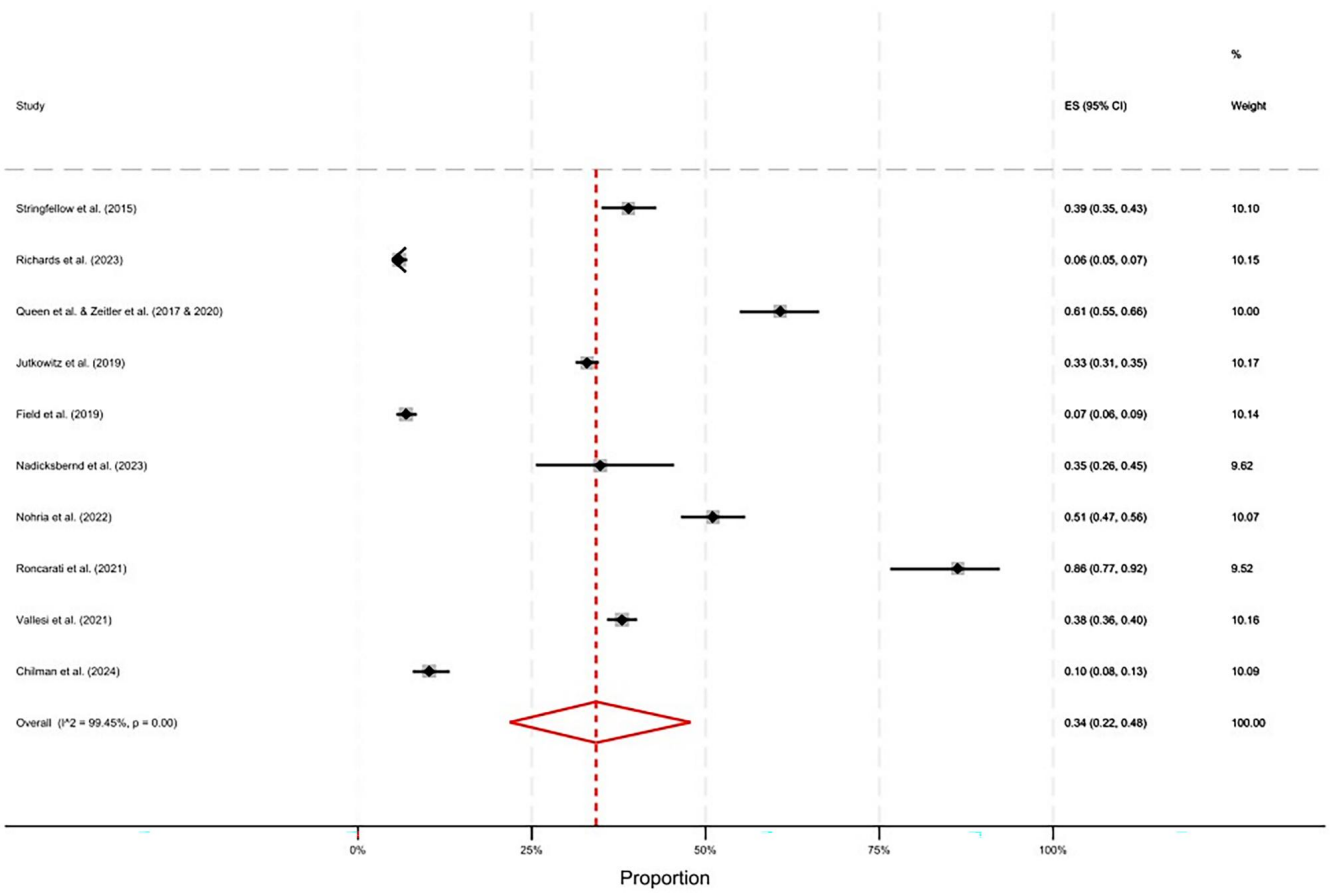
The pooled prevalence of trimorbidity for people with experience of homelessness, excluding non-probability sampling studies.

The pooled prevalence of multimorbidity for homeless populations was 45% (95% CI, 25–66) (Fig. 2). There was substantial heterogeneity between studies (*I*^2^ = 99.51%), which is likely to reflect the broad range of settings, countries, and conditions included in definitions of multimorbidity (Table 1), and is explored further in additional analyses. The pooled prevalence of trimorbidity was 34% (95% CI, 22–48) (Fig. 3). A range of prevalence estimates were reported across studies, depending on context. For example, Roncarati *et al.* [24] found a substantially higher prevalence of trimorbidity (86%) compared to other studies. This is likely because this study included people in a hostel for people with ‘complex needs’, while the other samples did not include this inclusion criteria for specialist services. On the other end of this spectrum, 3 studies found <10% prevalence of trimorbidity [36, 52]. One of these studies [52] only included young adults between the ages of 16 and 24, which is likely to explain this lower prevalence finding compared to other samples with older adults. One of these studies was also conducted with people who were formerly homeless and living in private households [20], who are likely to have better health compared to other samples with people currently homeless and using specialist services.

### Comparison studies

Six studies compared the prevalence of multimorbidity for people who experience homelessness compared to the general population in their statistical analysis. Two of these studies weighted or matched the non-homeless comparison samples to the age [42] and sex [32] characteristics of the homeless sample. The age-weighted study, by Rogans-Watson *et al.*, included people in a homeless hostel [42]. They found that all participants in the hostel had more long-term conditions than the mean number for people their age in the general population [42]. They also found that every homeless participant had more long-term conditions than average for 90-year-olds in the general population, despite the cohort’s average younger age of 56. The study by Nagy-Borsy *et al.* [32] weighted the general population estimates to both the age and sex characteristics of the homeless sample. They found the prevalence of multimorbidity in the homeless population to be 46.1%, compared to 14.3% in the general population, with a chi-squared test *P* values <.001. They also compared this to a 23.5% prevalence of multimorbidity in people in the general population who were in the lowest income quintile. Overall, both age- and sex-adjusted studies found a higher prevalence of multimorbidity in people who were homeless compared to the general population, and indications that people who experience homelessness experience premature multimorbidity in the life course.

The remaining four studies similarly reported on the prevalence of multimorbidity; however, prevalence estimates were not standardized by age or sex. Bensken *et al.* used mainstream healthcare practice records and found that patients who had homelessness recorded had a higher mean number of conditions (4.8, SD = 3.6) compared to people living in the highest areas of deprivation (3.3, SD = 3.3), and compared to the wider general population (2.6, SD = 2.8). This study had a substantially large sample size (total *N* = 1 974 766, homeless group *n* = 15 920) and was of high quality (Kmet risk of bias score = 0.86). Jutkowitz *et al.* [25] found a 6.9% prevalence of trimorbidity in stably housed veterans, compared to 33% for formerly homeless veterans. In a nationally representative private household survey study, Chilman *et al.* [20] compared people who were formerly homeless to people who had never been homeless. They found inequities for formerly homeless people for mental-physical multimorbidity (37.7%, compared to 12.1% in the never homeless group) and trimorbidity (8.9%, compared to 1.3% in the never homeless group). Lutchmun *et al.* [33], conversely to these other comparison studies, found a similar prevalence of multimorbidity in a securely housed group (44%) compared to a homeless group (45%). This study compared to people accessing humanitarian clinics which were provided to people without or with limited access to mainstream healthcare services, who are likely to have complex health needs.

### Additional analyses

Additional stratified meta-analyses were undertaken to explore possible sources of the high heterogeneity observed in Figs 2 and 3. Forest plots from these analyses are included in the Supplementary File S4. As expected, in age-stratified meta-analyses, studies with older participants found a higher prevalence of trimorbidity compared to studies conducted with younger age groups; this was not observed for multimorbidity; however only two studies had samples who were largely over the age of 50, thus limiting our conclusions. When stratified by method of morbidity ascertainment, self-reported multimorbidities tended to be more prevalent for multimorbidity but lower for trimorbidity; however, high levels of heterogeneity were observed. All but one of the samples were majority male, therefore a stratified analyses by sex could not be conducted.

We also conducted a meta-analysis looking at mental-physical multimorbidity only, which differs from multimorbidity as mental-physical multimorbidity requires at least one condition relating to physical health and at least one condition relating to mental health. The pooled prevalence of mental-physical multimorbidity was 54% (95% CI, 48–61), and the heterogeneity between studies was lower (*I*^2^ = 91.85%), however this was reported by few studies included in this review (*N* = 6 studies; see Supplementary Materials for more details).

## Discussion

This is the first systematic review to synthesize and appraise the existing evidence base on multimorbidity for people who have experienced homelessness. In our meta-analyses of homeless populations, when excluding studies which applied non-probability sampling strategies, there was a pooled prevalence of 45% (95% CI, 25–66) for multimorbidity and 34% (95% CI, 22–48) for trimorbidity. Previous research has found an estimated 23% prevalence of multimorbidity in the non-homeless general population, albeit within the specific context (Scotland) and methodology (electronic health records) [56]. While the totality of the evidence indicates severe inequalities in multimorbidity prevalence for people who experience homelessness, there was substantial heterogeneity between the included studies.

The prevalence of multimorbidity and trimorbidity varied greatly between studies, and *I*^2^ statistics indicated high levels of heterogeneity. We reported pooled effect statistics even where *I*^2^ values were high for ease of interpretation and due to the clinical relevance of results. However, the high level of heterogeneity means that these pooled estimates should be interpreted with caution. By stratifying analyses by sampling strategy, some patterns became evident (Supplementary File S4). When separating pooled estimates by sampling methods, we observed that multimorbidity prevalence estimates tended to be lower in studies which included the whole sampling frame compared to studies with convenience and snowball samples. This could be due to biases associated with non-probability sampling methods. When stratifying by method of morbidity ascertainment, prevalence estimates for multimorbidity tended to be lower in studies which sourced morbidities from a review of health records compared to self-report. This could be a consequence of under-diagnosis and/or under-recording in health record data. However, conversely the prevalence of trimorbidity was lower for studies where morbidities were self-reported by participants, and higher for studies involving a review of health records This could indicate recall and social desirability biases, which may be particularly relevant for mental health and substance use morbidities due to the stigmatization of these conditions [7]. Overall, the high heterogeneity between studies means that pooled estimates must be treated with caution, and prevalence estimates are likely impacted by factors of the study design. The large variety of methodological approaches in homelessness and multimorbidity research limits our ability to give reliable average proportions.

The link between homelessness and multimorbidity is likely a bi-directional relationship [57]. Research has shown that mental health and substance use problems tend to pre-date homelessness [58, 59] and that health problems increase the risk of becoming homeless in the first place [60]. Furthermore, homelessness has also been found to exacerbate and lead to mental and physical health problems [57, 59]. The findings from studies included in this systematic review indicated that health conditions accumulate for people who experience homelessness, with trimorbidity representing the most complex form of multimorbidity [3]. There was also evidence that multimorbidity occurs earlier in the life-course for people who experience homelessness compared to the general population [42]. These findings underscore the need for early and integrated interventions to support the health of people experiencing homelessness.

The findings of this systematic review suggest several evidence gaps in this area, particularly for people outside of services and for women who experience homelessness. Specialist homelessness services only represent a sub-group of the homeless population, and further research is required with homeless populations outside of these services.

Obtaining representative samples is extremely challenging in homelessness research as there is little information available on this population to construct a sampling frame [61], and because homelessness encompasses a range of experiences. Studies often sampled from specific areas. While a better understanding of local needs is important for service provision and commissioning, there is a paucity of nationally representative research, with the exception of a study recently conducted in England [20]. A variety of prevalence estimates for multimorbidity were found across specialist homeless services in the meta-analyses. This suggests that results from service and location-specific studies may be context-specific and demonstrates the limitations for the generalizability of this research.

Further studies which can make age and sex standardized comparisons with non-homeless populations would be of value. Only one study measured the prevalence of trimorbidity in the general population [20]. General population estimates of trimorbidity would enable a more comprehensive quantification of the scale of inequities for homeless populations, as would further conceptual work on trimorbidity and how this relates to quality of life and activities of daily living. Given the high heterogeneity between studies in this systematic review, future studies which use validated approaches to measuring multimorbidity [2, 10] and clear definitions of homelessness [62, 63] would provide a more consistent and reliable evidence-base.

The main strength of the systematic review lies in the thorough search strategy, which included 87 key word and subject heading search terms. Forward and backward citation searches were conducted, and authors of included papers were contacted to identify further research and reduce the risk of publication bias. Multiple reviewers were involved in the screening and data extraction, improving the overall quality and accuracy of the review. Studies were assessed using a risk of bias tool. However, it is a limitation that studies not in the English language were excluded. These findings cannot be generalized to lower- and middle-income countries, where the contextual factors related to homelessness differ from the high-income countries included in this review. Grey literature was not searched in the updated 2023 search as the Open Grey database was decommissioned, therefore studies outside of academic peer reviewed journals between 2021 and 2023 could have been missed. Contacting study authors for other relevant studies aimed to address this to some extent. Lastly, as discussed, the pooled prevalence estimates should be interpreted with the high levels of heterogeneity in mind.

To conclude, multimorbidity was found to be highly prevalent for people who experience homelessness. In meta-analyses, the prevalence of multimorbidity (45%) was double compared to estimates for the general population (23%) [56]. Approximately one-third of people who experience homelessness had trimorbid co-occurring conditions (34%), which encompassed mental, physical, and alcohol/substance use morbidities. For clinical practice and policies [64, 65], these findings highlight the importance of collaboration and co-ordination between multiple health services to support people experiencing homelessness.

## Supplementary Material

ckaf144_Supplementary_Data

## Data Availability

The data underlying this article will be shared on reasonable request to the corresponding author. Key points The literature in this area shows that people who experience homelessness experience a high prevalence of multimorbidity (co-occurring conditions; pooled prevalence estimate=45%), and trimorbidity (co-occurring mental, physical, and substance use conditions; pooled prevalence estimate=34%). The totality of the evidence shows severe inequalities in multiple mental and physical health conditions for people who experience homelessness compared to the general population; however, there was substantial heterogeneity between the included studies. Further research using validated approaches to measure multimorbidity, consistent definitions of homelessness, and research including women and those outside of specialist homelessness services, would improve the evidence-base. For clinical practice and policies, these findings highlight the importance of collaboration and co-ordination between multiple health services to support people experiencing homelessness.
