# Supplementary material for: The prevalence of multimorbidity with mental and physical health for people who experience homelessness: a systematic review
Source: Eur J Public Health. 2025 Aug 28;35(6):1170–7. doi: 10.1093/eurpub/ckaf144 (PMC12707478; doi:10.1093/eurpub/ckaf144)
Supplement: ckaf144_Supplementary_Data [file ckaf144_supplementary_data.docx]

**Supplementary Materials**

Contents

[**1.** **Database search strategy** 2](#_Toc195781883)

[**2.** **Details of included studies** 4](#_Toc195781884)

[**3.** **Risk of bias assessments** 12](#_Toc195781885)

[**4.** **Meta-analyses forest plots** 13](#_Toc195781886)

[**5.** **References for Supplementary Materials** 20](#_Toc195781887)

[**6.** **References for Main Paper** 21](#_Toc195781888)

[**7.** **PRISMA** 23](#_Toc195781889)

# **Database search strategy**

The search terms for the systematic review, developed in Medline in 2021 and repeated in 2023 and 2025, are as follows:

**Search Strategies**

**Medline**

Database: Ovid MEDLINE(R) ALL <1946 to April 22, 2021>

Search Strategy:

--------------------------------------------------------------------------------

1 exp vulnerable populations/ (11472)

2 exp prisoners/ (17209)

3 exp homeless persons/ (9420)

4 exp sex workers/ (2366)

5 exp prostitution/ (6124)

6 prison*.mp. (29896)

7 imprison*.mp. (2593)

8 homeless*.mp. (13926)

9 "temporary hous*".mp. (199)

10 "rough sleep*".mp. (51)

11 "street-health ".mp. (22)

12 "sex work*".mp. (10494)

13 prostitut*.mp. (4143)

14 incarcerat*.mp. (12415)

15 jail*.mp. (3783)

16 1 or 2 or 3 or 4 or 5 or 6 or 7 or 8 or 9 or 10 or 11 or 12 or 13 or 14 or 15 (75187)

17 exp Multimorbidity/ (1289)

18 multimorbid*.mp. (6235)

19 multi-morbid*.mp. (922)

20 "multiple morbidity*".mp. (128)

21 "intercurrent morbidity".mp. (5)

22 "co?existing condition*".mp. (583)

23 "co?existing diagnos*".mp. (64)

24 "co?existing disease*".mp. (780)

25 "co?existing illness*".mp. (194)

26 "co?existing health problem*".mp. (11)

27 "co?existing patholog*".mp. (188)

28 "co?occurring condition*".mp. (11)

29 "co?occurring diagnos*".mp. (3)

30 "co?occurring disease*".mp. (0)

31 "co?occurring health problem*".mp. (0)

32 "co?occurring illness*".mp. (1)

33 "co?occurring patholog*".mp. (0)

34 "concurrent condition*".mp. (272)

35 "concurrent diagnos*".mp. (558)

36 "concurrent disease*".mp. (1023)

37 "concurrent illness*".mp. (354)

38 "concurrent health problem*".mp. (19)

39 "concurrent patholog*".mp. (148)

40 "multiple chronic disease*".mp. (507)

41 "several chronic disease*".mp. (1083)

42 "multiple health problem*".mp. (192)

43 "several health problem*".mp. (177)

44 "one or more chronic conditions".mp. (119)

45 "multiple illness*".mp. (151)

46 "multiple diagnos*".mp. (1218)

47 "multiple disease*".mp. (3612)

48 "multiple patholog*".mp. (1488)

49 "multiple condition*".mp. (1254)

50 "multiple health problem*".mp. (192)

51 multi?disease*.mp. (57)

52 multi?patholog*.mp. (18)

53 pluri?patholog*.mp. (42)

54 poly?patholog*.mp. (186)

55 "long?term condition*".mp. (9)

56 "mental health".mp. (209865)

57 "physical health".mp. (23096)

58 56 and 57 (9416)

59 exp comorbidity/ (115595)

60 co?morbid*.mp. (247710)

61 "associated condition*".mp. (3407)

62 "associated diagnos*".mp. (732)

63 "associated disease*".mp. (14483)

64 "associated health problem*".mp. (211)

65 "associated illness*".mp. (986)

66 "associated morbid*".mp. (5919)

67 "associated patholog*".mp. (3900)

68 17 or 18 or 19 or 20 or 21 or 22 or 23 or 24 or 25 or 26 or 27 or 28 or 29 or 30 or 31 or 32 or 33 or 34 or 35 or 36 or 37 or 38 or 39 or 40 or 41 or 42 or 43 or 44 or 45 or 46 or 47 or 48 or 49 or 50 or 51 or 52 or 53 or 54 or 55 or 58 or 59 or 60 or 61 or 62 or 63 or 64 or 65 or 66 or 67 (301590)

69 exp Mental Disorders/ (1279989)

70 exp Mental Health/ (42767)

71 "mental health".mp. (209865)

72 "mental illness*".mp. (33998)

73 "mental disorder*".mp. (206373)

74 "mental disease*".mp. (3617)

75 "mental problem*".mp. (1136)

76 "severe mental illness*".mp. (4999)

77 "severe mental disorder*".mp. (1457)

78 psychiat*.mp. (366738)

79 schiz*.mp. (178012)

80 psychosis.mp. (40467)

81 psychotic.mp. (69872)

82 depress*.mp. (569184)

83 bipolar.mp. (82229)

84 "personality disorder*".mp. (46391)

85 "substance misuse".mp. (2832)

86 "substance use disorder".mp. (6743)

87 anxiety.mp. (253652)

88 "intellectual disab*".mp. (66328)

89 autis*.mp. (56192)

90 "common mental illness*".mp. (207)

91 "common mental disorder*".mp. (2885)

92 69 or 70 or 71 or 72 or 73 or 74 or 75 or 76 or 77 or 78 or 79 or 80 or 81 or 82 or 83 or 84 or 85 or 86 or 87 or 88 or 89 or 90 or 91 (2030231)

93 16 and 68 and 92 (2042)

94 limit 93 to (english language and humans and yr="1997 -Current") (1617)

# **Table S1: Details of included studies**

| Author/s and year | Country | Study design & sampling strategy | Setting | N homeless | Multimorbidity outcome(s) | Number of morbidities | Method of morbidity ascertainment | Multimorbidity prevalence/risk outcome |
| --- | --- | --- | --- | --- | --- | --- | --- | --- |
| Stringfellow et al. (2015) | United States | Cross-sectional, probability sampling | Specialist primary care services | 601 | Trimorbidity | 20 | Self-report and structured validated scales | 39% trimorbidity prevalence |
| Richards et al. (2023) | United States | Repeated cross-sectional, probability sampling | Unsheltered young adults | 1672 | Trimorbidity | 10 | Self-report | 6% trimorbidity weighted prevalence estimate |
| Chilman et al. (2024) | England | Cross-sectional, probability sampling | People living in private households | 535 | Mental-physical multimorbidity; trimorbidity | 24 | Self-report and structured validated scales | 38% mental-physical multimorbidity survey-weighted prevalence estimate; 9% trimorbidity survey-weighted prevalence estimate |
| Queen et al. (2017)  &  Zeitler et al. (2020)^1^ | Scotland | Cross-sectional, samples all patients in services | Specialist primary care services | Glasgow n=133  Edinburgh n=150 | Multimorbidity; mental-physical multimorbidity; trimorbidity | 40 | Review of health record | Glasgow:  >75% multimorbidity prevalence;  61% mental-physical multimorbidity prevalence; 49% trimorbidity prevalence; median number of conditions=6 (SD=3.9)  Edinburgh: 71% trimorbidity prevalence; median number of conditions=7 (SD 3.3) |
| Bowen et al. (2019) | England | Cross-sectional, samples all patients in service | Specialist primary care service | 928 | Multimorbidity | 21 | Review of health record | 21% multimorbidity prevalence |
| Jutkowitz et al. (2019) | United States | Cross-sectional, samples all patients in service | Veterans admitted to nursing home (homeless in year prior to admission) | 3355 | Trimorbidity | 13 | Review of health record | 33% trimorbidity prevalence; adjusted relative risk=2.57 (95% CI 2.40-2.74) |
| Field et al. (2019) | England | Retrospective service evaluation, samples all patients in services | Specialist inpatient services | 1135 | Mental-physical multimorbidity; substance-physical multimorbidity; trimorbidity | No limits | Review of health record | 56% multimorbidity prevalence  Mutually exclusive groups:  7% mental-physical multimorbidity; 40% substance-physical multimorbidity; 2% mental-substance multimorbidity; 7% trimorbidity |
| Bensken et al. (2021) | United States | Cross-sectional, samples all patients in services | Outpatient mainstream healthcare services | 15,920 | Multimorbidity | 31 | Review of health record | Mean number of conditions=4.8 (SD=3.6); median number of conditions=4 (ICR 2-7) |
| Lutchmun et al. (2022) | Germany | Cross-sectional, samples all patients in services | Humanitarian health clinics | 333 | Multimorbidity | No limits | Review of health record | 45% multimorbidity prevalence; association of multimorbidity with secure housing (compared to homeless group) Odds Ratio=1.08 (95% CI 0.55-2.11) |
| Plezia et al. (2023) | United States | Retrospective review of records, samples all homeless patients from needs assessment | Mainstream emergency department service | 57 | Multimorbidity | No limits | Review of health record | 56% prevalence of multimorbidity |
| Seto et al. (2024) | United States | Retrospective review of records, samples all patients in service | Specialist homelessness healthcare service | 526 | Multimorbidity | No limits | Review of health record | 9% prevalence of multimorbidity |
| Madigan & Friedman (2021) | United States | Cross-sectional, non-probability sampling | Outpatient and inpatient mainstream services | Not available^3^ | Multimorbidity^4^ | 31 | Review of health record per service visit | Outpatient service: 17% multimorbidity prevalence, mean number of conditions=1.37 (SD 1.33)  Inpatient service: 48% multimorbidity prevalence, mean number of conditions=3.07 (SD 1.68) |
| Nadicksbernd et al. (2023) | England | Cross-sectional, non-probability sampling | Specialist inpatient services | 86 | Multimorbidity; trimorbidity; mental-physical multimorbidity; physical-substance multimorbidity | No limits | Reported by hospital team | 72% multimorbidity prevalence  Non-mutually exclusive groups:  35% trimorbidity; 48% mental-physical multimorbidity; 59% physical-substance multimorbidity |
| Nohria et al. (2022) | United States | Programme evaluation, non-probability sampling | Transitional care programme^2^ | 450 | Mental-physical multimorbidity; substance-physical multimorbidity; trimorbidity | 33 | Review of health record | Non-mutually exclusive groups:  64% mental-physical multimorbidity prevalence; 66% substance-physical multimorbidity prevalence; 51% trimorbidity prevalence |
| Roncarati et al. (2021) | United States | Longitudinal open-cohort, non-probability sampling | Permanent supported housing | 73 | Trimorbidity | No limits | Review of health record | 86% trimorbidity |
| Vallesi et al. (2021) | Australia | Retrospective cohort, non-probability sampling | Specialist primary care service | 2068 | Multimorbidity; mental-physical multimorbidity; trimorbidity | 40 | Review of health record | 75% multimorbidity prevalence; mean number of conditions=3.3 (SD 2.4)  Non-mutually exclusive groups:  51% mental-physical multimorbidity prevalence; 48% mental-substance multimorbidity prevalence; 47% physical-substance multimorbidity; 38% trimorbidity prevalence |
| Kaushal et al. (2021) | England | Retrospective cross-sectional, non-probability sampling | Mainstream primary care services within a region in the top decile of area-level deprivation | 43 | Multimorbidity | 22 | Review of health record | 35% multimorbidity prevalence |
| Keogh et al. (2015) | Ireland | Cross-sectional, non-probability sampling | Specialist primary care service | 105 | Multimorbidity | 41 | Self-report and structured validated scales | 84% prevalence of multimorbidity; mean number of conditions=3.3 (range 1-11) |
| Henwood et al. (2017) | United States | Cross-sectional, non-probability sampling | Permanent supported housing | 421 | Multimorbidity | 11 | Self-report | 90% multimorbidity prevalence |
| Vila-Rodriguez et al. (2013)  &  Barbic et al. (2018)^5^ | Canada | Prospective cohort (baseline)^6^, non-probability sampling | Single-room occupancy hotels | 293 | Multimorbidity | 12 | Structured interview (self-report), reviews of hospitalization records, clinical interviews, MRI scans, urine and blood tests | Median number of conditions=3 (IQR 2-4)  Median number of conditions for sub-sample aged 18-29=2 (IQR 1-3) |
| Vickery et al. (2021) | United States | Repeated cross-sectional^3^, non-probability sampling | Emergency and domestic violence shelters; transitional housing programmes; encampments; specialist outreach services; people living with friends/family | 4,181^7^ | Bi-morbidity^8^; trimorbidity | 12 | Self-report | 32% bi-morbidity prevalence; 16% trimorbidity prevalence |
| Roche et al. (2018) | Australia | Cross-sectional, non-probability sampling | Specialist primary care service | 40 | Multimorbidity | 13 | Self-report | 88% multimorbidity prevalence |
| Rogans-Watson et al. (2020) | England | Cross-sectional, non-probability sampling | Hostel for single homeless people above 30 years old who have complex needs | 33 | Multimorbidity | 39 | Self-report | 100% |
| Nagy-Borsy et al. (2021) | Hungary | Cross-sectional, non-probability sampling | Shelters; specialist health services; temporary accommodation | 453 | Multimorbidity | No limits | Self-report; review of health record (where available) | 46% multimorbidity prevalence |
| Lowrie et al. (2023)  &  Jones et al. (2023) | Scotland | Randomised Controlled Trial (baseline)^6^, non-probability sampling | Temporary accommodation services; low threshold city centre venues; people on the street | 128 | Multimorbidity | No limits | Self-report; review of health record | 100% multimorbidity prevalence  (2-4 conditions=9%; 5-8 conditions=46%; 9-16 conditions=45%) |
| Shulman et al. (2023) | England | Cross-sectional, non-probability sampling | Hostels | 2355 | Trimorbidity | No limits | Reported by hostel manager | 31% trimorbidity prevalence |
| van Everdingen et al. (2021) | Netherlands | Cross-sectional, non-probability sampling | Specialist homelessness services^9^ | 436 | Multimorbidity^10^ | 24 | Self-report and | Non-mutually exclusive groups: 95% multimorbidity; 59% mental-physical multimorbidity; 78% mental-substance multimorbidity; 45% physical-substance multimorbidity |
| Perna et al. (2024) | United States | Longitudinal study, non-probability | Specialist homelessness healthcare service | 118 | Multimorbidity | No limits | Self-report | 39% multimorbidity prevalence: 16% prevalence of two conditions, 23% prevalence of 3 or more conditions |
| Levitt et al. (2009) | United States | Cross-sectional, non-probability | Rough sleepers on streets, in parks, publicly accessible outdoor areas, and transit hubs | 1,093 | Trimorbidity | No limits | Self-report | 14% trimorbidity prevalence |
| Nicholson et al. (2010) | Canada | Cross-sectional, non-probability | Rough sleepers on streets | 137 | Multimorbidity | 13 | Self-report | 55% multimorbidity prevalence |
| *^1^These two studies were combined as they used the same primary sample in Glasgow (n=133); Zeitler and colleagues compared the sample in Glasgow with a new additional sample in Edinburgh (n=150).*  *^2^This programme provided support for people experiencing housing insecurity when discharged from acute care facilities.*  *^3^In this study, there were 154,173 patient visits to the services, but one patient could have multiple visits.*  *^4^While other studies defined multimorbidity as 2 or more conditions, this study defined multimorbidity as 3 or more conditions.*  *^5^These two studies were combined as they used the same primary sample (n=293); Barbic and colleagues limited the sample to participants aged 18-29 years only (n=101).*  *^6^Multimorbidity was measured cross-sectionally (at baseline).*  *^7^Across 7 cross-sectional surveys (conducted between 2000 and 2018), there were 25,552 responses. However, as some participants may be included in multiple responses, the sample size is unknown. We have therefore taken the number of responses and multimorbidity data from the latest survey year (2018).*  *^8^Bi-morbidity was defined as conditions in two domains out of: physical health, mental health, or substance use disorders.*  *^9^Includes shelters and other services.*  *^10^Multimorbidity was defined as co-occurring conditions between four domains: physical conditions, mental illness, intellectual impairments, and addiction.* | | | | | | | | |

# **Risk of bias assessments**

Table S2: Kmet risk of bias^1^ scores for studies applying probability sampling strategies or those including the whole sampling frame (for example, electronic health record studies).

| Study author name(s), year | Kmet risk of bias assessment score |
| --- | --- |
| Stringfellow et al. (2015) | 0.55 |
| Richards et al. (2023) | 0.85 |
| Queen et al. (2017) & Zeitler et al. (2020) | 0.70 |
| Bowen et al. (2019) | 0.75 |
| Jutkowitz et al. (2019) | 0.90 |
| Field et al. (2019) | 0.80 |
| Bensken et al. (2021) | 0.86 |
| Lutchmun et al. (2022) | 0.81 |
| Madigan & Friedman (2021) | 0.65 |
| Nadicksbernd et al. (2023) | 0.45 |
| Nohria et al. (2022) | 0.85 |
| Roncarati et al. (2021) | 0.70 |
| Vallesi et al. (2021) | 0.90 |
| Kaushal et al. (2021) | 0.60 |
| Chilman et al. (2024) | 0.90 |
| Plezia et al. (2023) | 0.65 |
| Seto et al. (2024) | 0.70 |
| Average score (Standard Deviation): | **0.74 (0.14)** |

Table S3: Kmet risk of bias^1^ scores for studies applying non-probability sampling strategies.

| Study author name(s), year, and reference | Kmet risk of bias assessment score |
| --- | --- |
| Keogh et al. (2015) | 0.65 |
| Henwood et al. (2017) | 0.55 |
| Vila-Rodriguez et al. (2013) & Barbic et al. (2018) | 0.70 |
| Vickery et al. (2021) | 0.75 |
| Roche et al. (2018) | 0.55 |
| Rogans-Watson et al. (2020) | 0.62 |
| Nagy-Borsy et al. (2021) | 0.57 |
| Lowrie et al. (2023) & Jones et al. (2023) | 0.65 |
| Shulman et al. (2023) | 0.45 |
| van Everdingen et al. (2021) | 0.50 |
| Perna et al. (2024) | 0.60 |
| Levitt et al. (2009) | 0.70 |
| Nicholson et al. (2010) | 0.75 |
| Jones et al. (2023) | 0.65 |
| Average score (Standard Deviation): | **0.62 (0.09)** |

# **Meta-analyses forest plots**

**Stratified by sampling strategy**

The meta-analyses in the main paper includes studies which applied probability sampling measures or included the whole sampling frame (for example, electronic health record studies of all patients in a homelessness health service). The below forest plots include all studies which investigated the outcome, stratified by sampling strategy.

Figure S1: Multimorbidity proportional meta-analysis forest plot, stratified by sampling strategy.

**
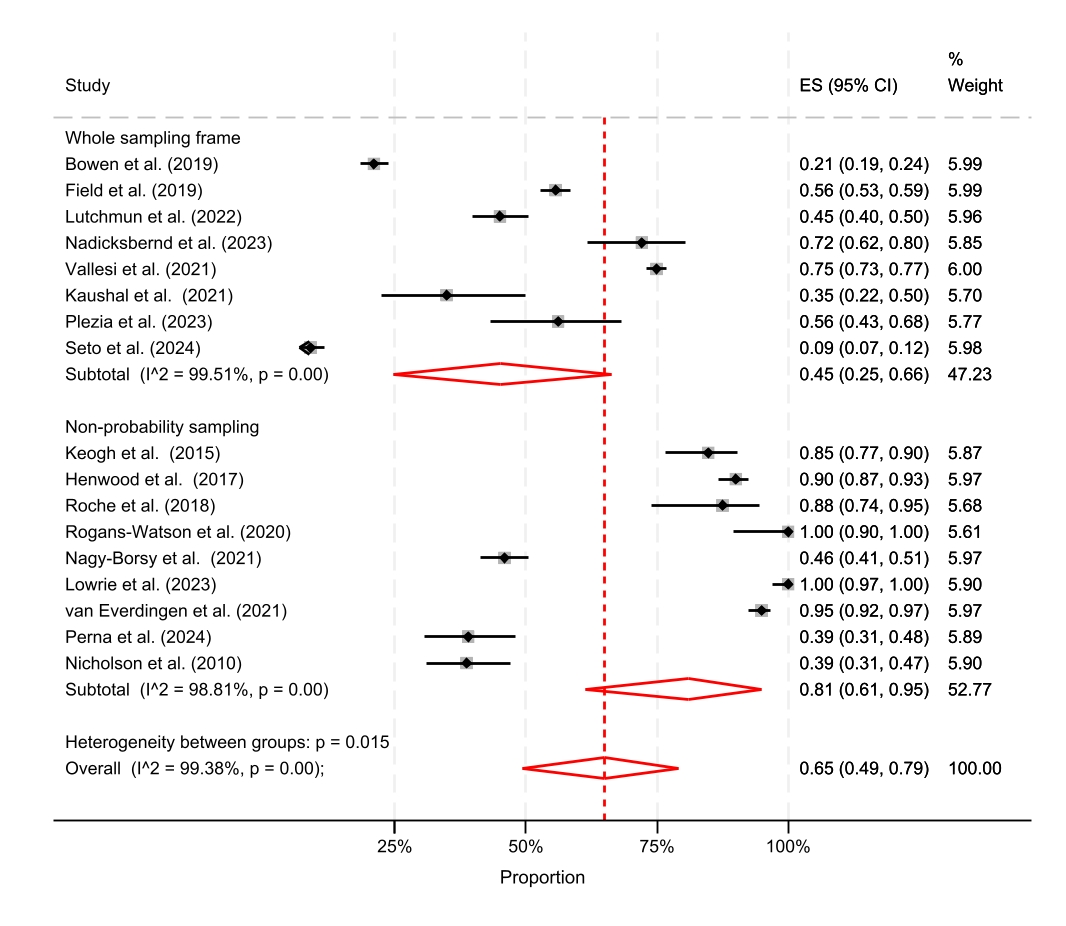
**

*Figure S2: Trimorbidity proportional meta-analysis forest plot, stratified by sampling strategy.*


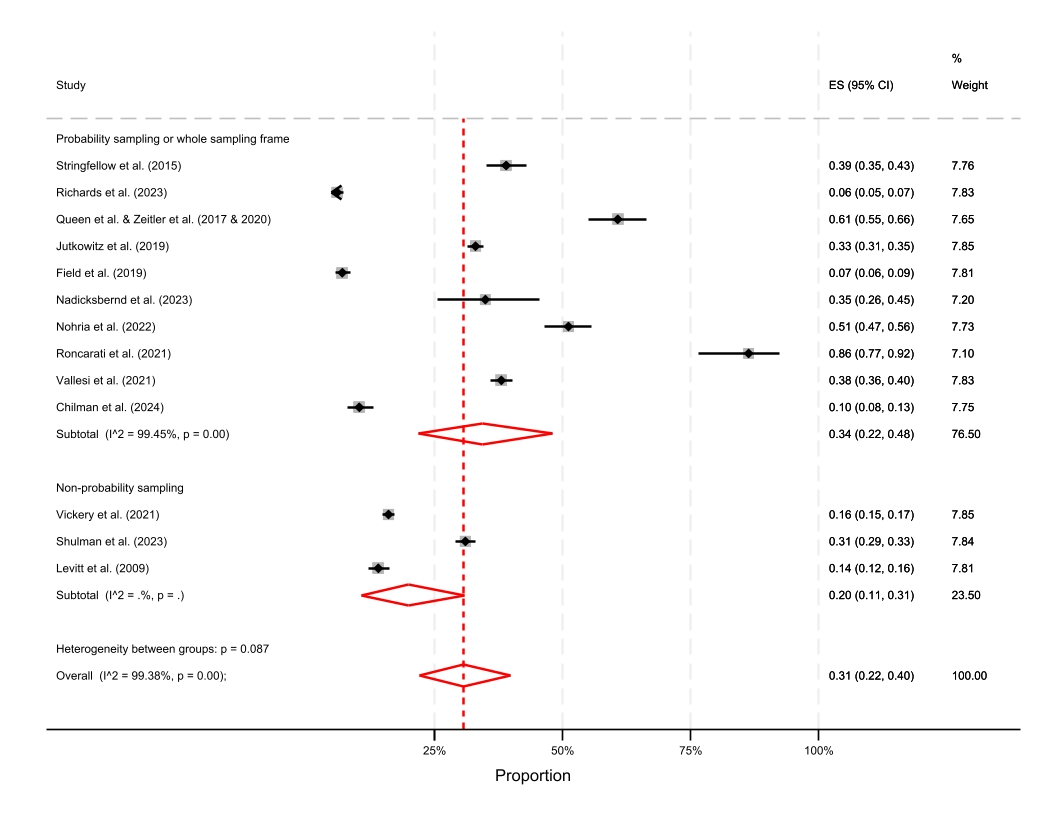


**Stratified by method of multimorbidity ascertainment**

Further sub-group analyses by method of morbidity ascertainment aimed to explore possible reasons underlying substantial heterogeneity in estimates for multimorbidity and trimorbidity prevalence. Studies were grouped into those which ascertained morbidities using (1) health records, (2) self-report, or (3) a combination of health records, self-report, and/or other methods.


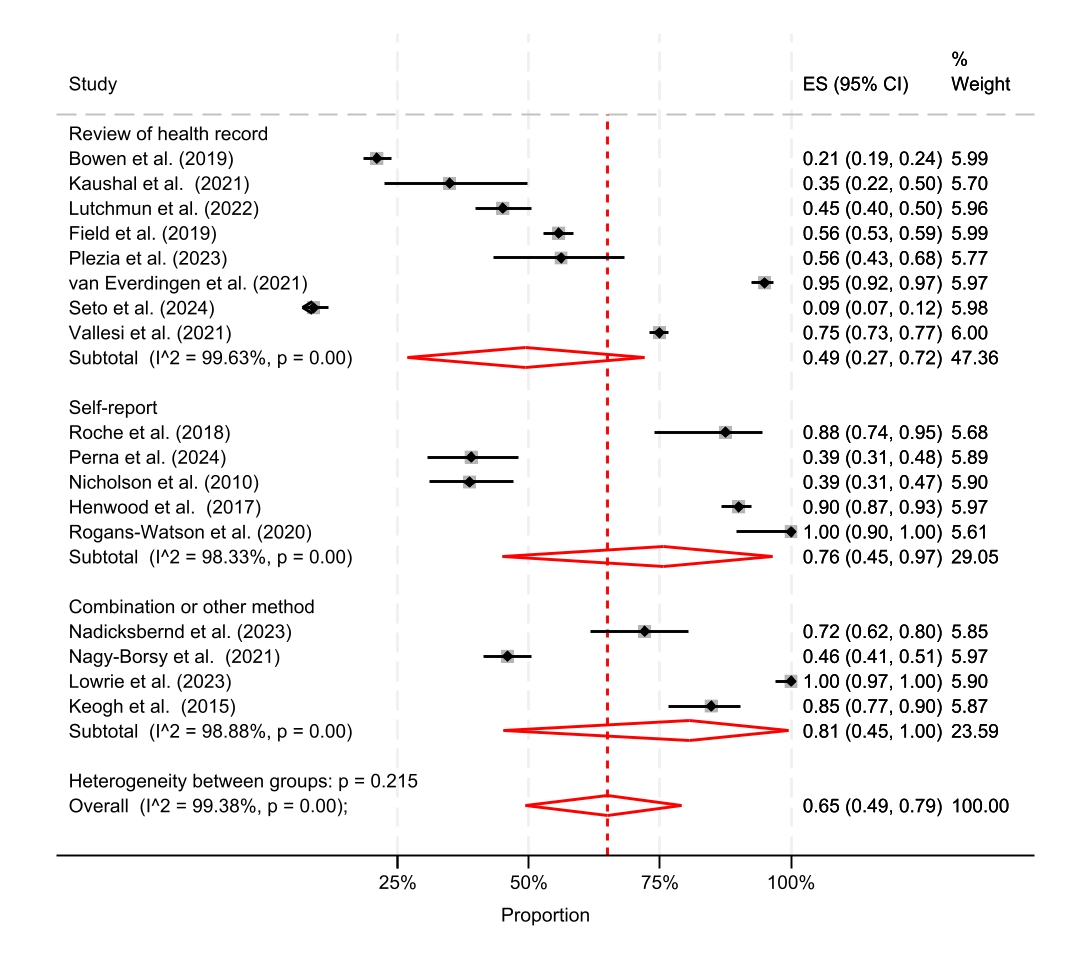
*Figure S3: Multimorbidity proportional meta-analysis forest plot, stratified by method of morbidity ascertainment.*


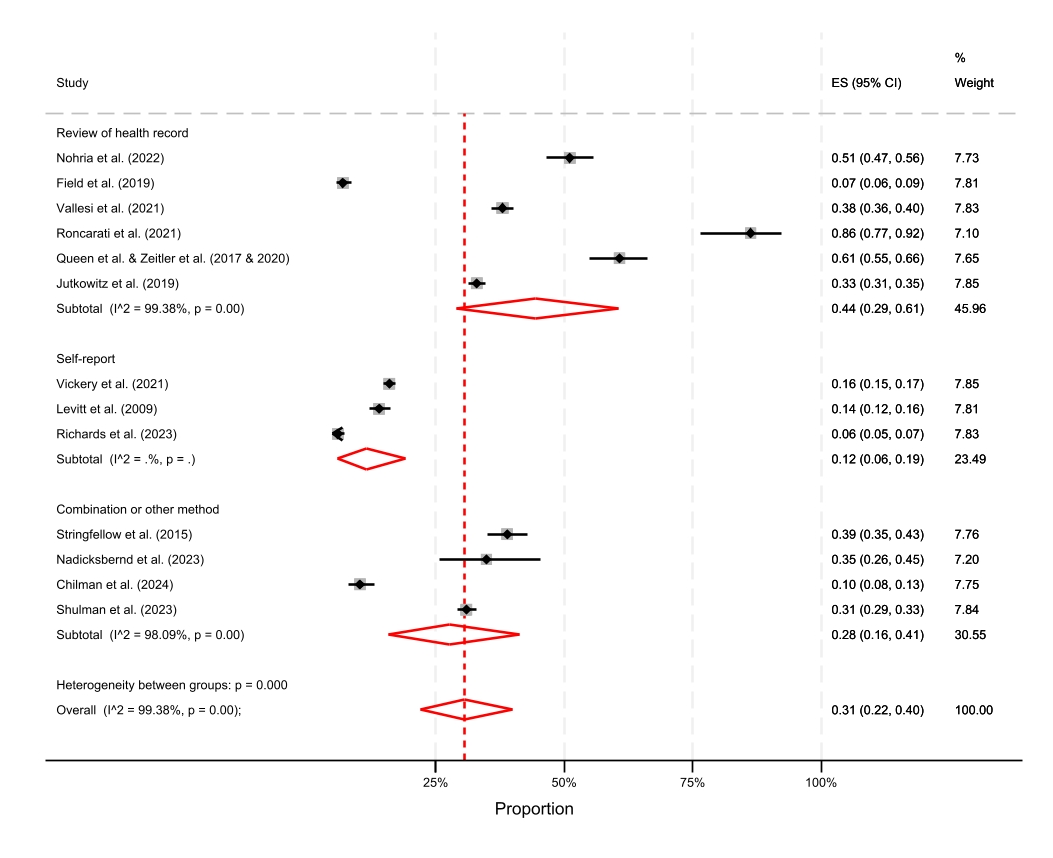
*Figure S4: Trimorbidity proportional meta-analysis forest plot, stratified by method of morbidity ascertainment.*

**Stratified by age**

Studies which employed non-probability sampling strategies were excluded prior to stratification by age. Studies were then grouped into those which included homeless samples who were either by majority or on average under the age of 50, and those where samples were majority or on average aged 50 and above. This age cut-off was chosen based on previous multimorbidity studies^2,3^.

Figure S5: Multimorbidity proportional meta-analysis forest plot, stratified by age and excluding non-probability sampling studies.


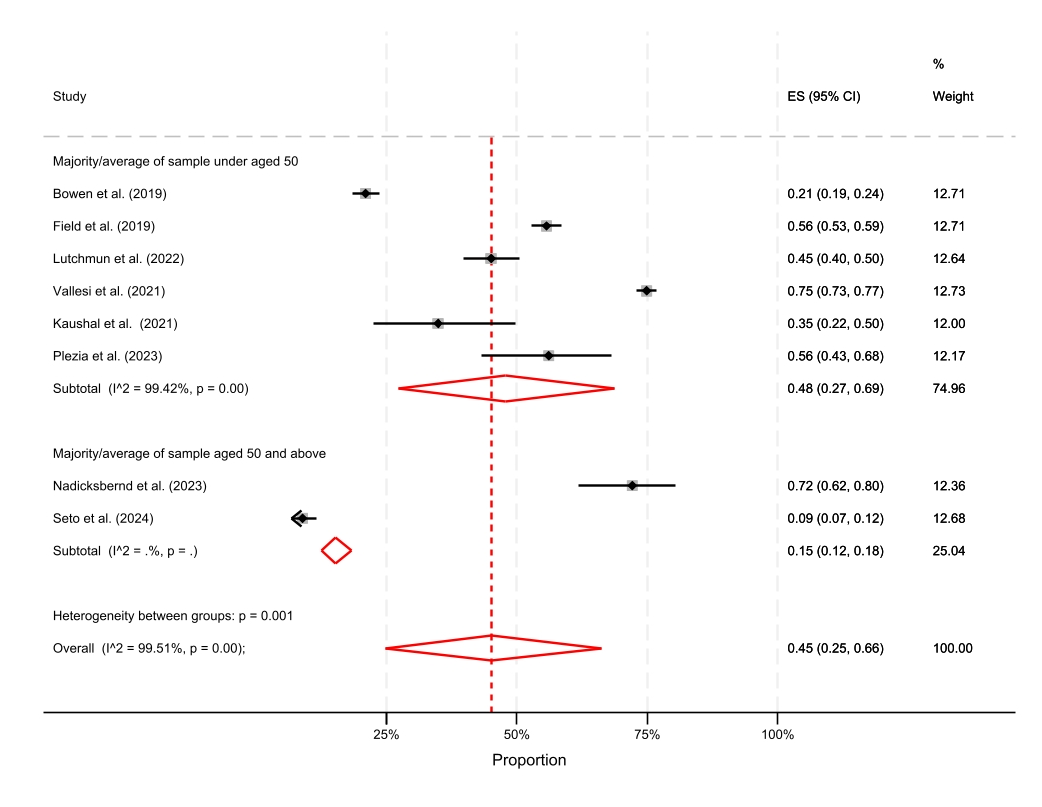


Figure S6: Trimorbidity proportional meta-analysis forest plot, stratified by age and excluding non-probability sampling studies.


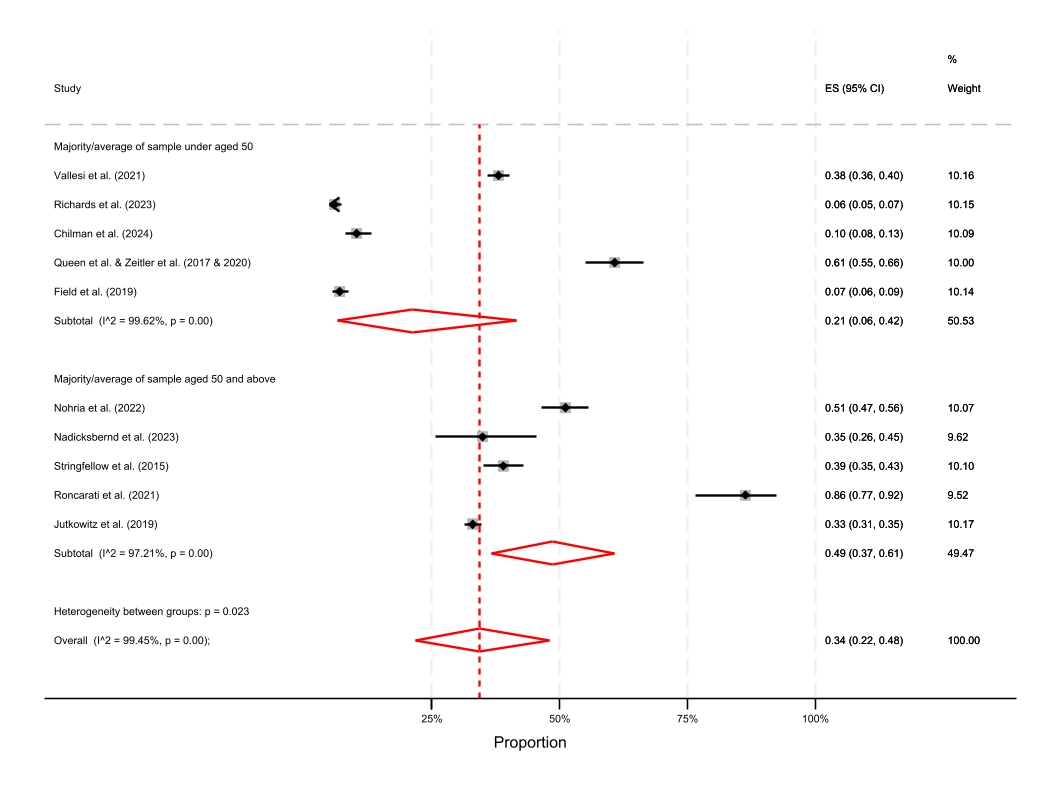


**Mental-physical multimorbidity**

Mental-physical multimorbidity is defined as at least two co-occurring conditions where at least one condition relates to physical health and at least one condition relates to mental health. This differs from multimorbidity which may include any combination of mental and/or physical morbidities, as presented in the main paper.

Figure S7: Multimorbidity proportional meta-analysis forest plot, stratified by sampling strategy.


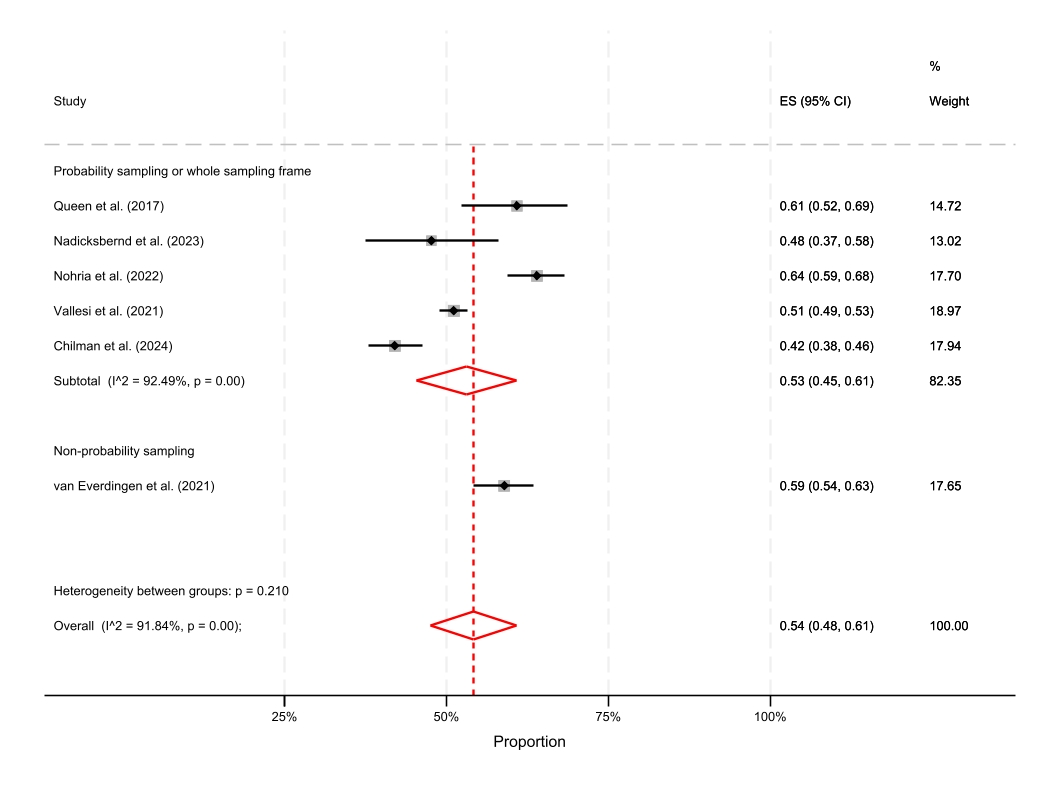


# **References for Supplementary Materials**

1. Kmet LM, Cook LS, Lee RC. Standard quality assessment criteria for evaluating primary research papers from a variety of fields. 2004;

2. Barnett K, Mercer SW, Norbury M, Watt G, Wyke S, Guthrie B. Epidemiology of multimorbidity and implications for health care, research, and medical education: a cross-sectional study. *The Lancet*. 2012;380(9836):37-43.

3. Marengoni A, Angleman S, Melis R, et al. Aging with multimorbidity: a systematic review of the literature. *Ageing research reviews*. 2011;10(4):430-439.

# **References for Main Paper**

41. Vickery KD, Winkelman TN, Ford BR, et al. Trends in trimorbidity among adults experiencing homelessness in minnesota, 2000-2018. Medical care. 2021;59(Suppl 2):S220.

42. Rogans-Watson R, Shulman C, Lewer D, Armstrong M, Hudson B. Premature frailty, geriatric conditions and multimorbidity among people experiencing homelessness: a cross-sectional observational study in a London hostel. Housing, Care and Support. 2020;

43. Lowrie R, McPherson A, Mair FS, et al. Baseline characteristics of people experiencing homelessness with a recent drug overdose in the PHOENIx pilot randomised controlled trial. Harm reduction journal. 2023;20(1):46.

44. Shulman C, Nadicksbernd J, Nguyen T, et al. People living in homeless hostels: a survey of health and care needs. Clinical Medicine. 2023;23(4):387-394.

45. Everdingen Cv, Peerenboom PB, Velden Kvd, Delespaul P. Health patterns reveal interdependent needs of Dutch Homeless Service Users. Frontiers in Psychiatry. 2021;12:614526.

46. Perna LK, Patterson C, Chairez AA, et al. Healthcare outcomes for unsheltered persons experiencing homelessness using a street medicine model of care: a pilot program evaluation. Cogent Social Sciences. 2024;10(1):2328895.

47. Seto BK, Singh DS, Seto JC, et al. Describing the Medical Needs of Hawai ‘i’s Houseless Population During COVID at Free Student Run Outpatient Clinics (Hawai ‘i HOME Project). Hawai'i Journal of Health & Social Welfare. 2024;83(6):158.

48. Henwood BF, Lahey J, Rhoades H, Winetrobe H, Wenzel SL. Examining the health status of homeless adults entering permanent supportive housing. Journal of public health. 2018;40(2):415-418.

49. Bensken WP, Krieger NI, Berg KA, Einstadter D, Dalton JE, Perzynski AT. Health status and chronic disease burden of the homeless population: an analysis of two decades of multi-institutional electronic medical records. Journal of health care for the poor and underserved. 2021;32(3):1619.

50. Kaushal R, Jagpal P, Khanal S, et al. Representation of persons experiencing homelessness and coding of homelessness in general practices: descriptive evaluation using healthcare utilisation data. BJGP open. 2021;5(4)

51. Plezia A, Renna D, Feijoo M, Feffer M, Nguyen T. 176 A Preliminary Assessment of Emergency Department Utilization and Trends by Patients Experiencing Homelessness. Annals of Emergency Medicine. 2023;82(4):S79-S80.

52. Richards J, Henwood BF, Porter N, Kuhn R. Examining the Role of Duration and Frequency of Homelessness on Health Outcomes Among Unsheltered Young Adults. Journal of Adolescent Health. 2023;

53. Levitt AJ, Culhane DP, DeGenova J, O'quinn P, Bainbridge J. Health and social characteristics of homeless adults in Manhattan who were chronically or not chronically unsheltered. Psychiatric Services. 2009;60(7):978-981.

54. Nicholson CL, Graham JR, Emery JH, Schiff JW, Giacomin ML, Tanasescu AI. Describing the health of the absolutely homeless population in downtown Calgary 2008. Canadian Journal of Urban Research. 2010;19(2):62-79.

55. Elixhauser A, Steiner C, Harris DR, Coffey RM. Comorbidity measures for use with administrative data. Medical care. 1998:8-27.

56. Barnett K, Mercer SW, Norbury M, Watt G, Wyke S, Guthrie B. Epidemiology of multimorbidity and implications for health care, research, and medical education: a cross-sectional study. The Lancet. 2012;380(9836):37-43.

57. Fazel S. The bidirectional association between psychiatric disorders and sheltered homelessness. 2023;

58. Fitzpatrick S, Bramley G, Johnsen S. Pathways into multiple exclusion homelessness in seven UK cities. Urban Studies. 2013;50(1):148-168.

59. Pattison B, McCarthy L. The role of mental health in multiple exclusion homelessness. Social Policy and Society. 2022;21(3):405-421.

60. Nilsson SF, Nordentoft M, Hjorthøj C. Individual-level predictors for becoming homeless and exiting homelessness: a systematic review and meta-analysis. Journal of urban health. 2019;96:741-750.

61. Burnam MA, Koegel P. Methodology for obtaining a representative sample of homeless persons: the Los Angeles Skid Row Study. Evaluation review. 1988;12(2):117-152.

62. Amore K, Baker M, Howden-Chapman P. The ETHOS definition and classification of homelessness: an analysis. European Journal of Homelessness. 2011;5(2)

63. Edgar B. The ETHOS definition and classification of homelessness and housing exclusion. European Journal of Homelessness. 2012;6(2):219-225.

64. National Institute for Health and Care Excellence. Integrated health and social care for people experiencing homelessness. 2022;

65. Clifford B, Wood L, Vallesi S, et al. Integrating healthcare services for people experiencing homelessness in Australia: key issues and research principles. Integrated Healthcare Journal. 2022;4(1):e000065.

# **PRISMA**

| **Section and Topic** | **Item #** | **Checklist item** | **Location where item is reported** |
| --- | --- | --- | --- |
| **TITLE** | | |  |
| Title | 1 | Identify the report as a systematic review. | Page 1 |
| **ABSTRACT** | | |  |
| Abstract | 2 | See the PRISMA 2020 for Abstracts checklist. | Page 2 |
| **INTRODUCTION** | | |  |
| Rationale | 3 | Describe the rationale for the review in the context of existing knowledge. | Pages 4-5 |
| Objectives | 4 | Provide an explicit statement of the objective(s) or question(s) the review addresses. | Page 5 |
| **METHODS** | | |  |
| Eligibility criteria | 5 | Specify the inclusion and exclusion criteria for the review and how studies were grouped for the syntheses. | Page 6 |
| Information sources | 6 | Specify all databases, registers, websites, organisations, reference lists and other sources searched or consulted to identify studies. Specify the date when each source was last searched or consulted. | Pages 5-6, Figure 1 |
| Search strategy | 7 | Present the full search strategies for all databases, registers and websites, including any filters and limits used. | Supplementary material 1 |
| Selection process | 8 | Specify the methods used to decide whether a study met the inclusion criteria of the review, including how many reviewers screened each record and each report retrieved, whether they worked independently, and if applicable, details of automation tools used in the process. | Pages 5-6, Figure 1 |
| Data collection process | 9 | Specify the methods used to collect data from reports, including how many reviewers collected data from each report, whether they worked independently, any processes for obtaining or confirming data from study investigators, and if applicable, details of automation tools used in the process. | Pages 6-7 |
| Data items | 10a | List and define all outcomes for which data were sought. Specify whether all results that were compatible with each outcome domain in each study were sought (e.g. for all measures, time points, analyses), and if not, the methods used to decide which results to collect. | Page 6 |
|  | 10b | List and define all other variables for which data were sought (e.g. participant and intervention characteristics, funding sources). Describe any assumptions made about any missing or unclear information. | Page 6 |
| Study risk of bias assessment | 11 | Specify the methods used to assess risk of bias in the included studies, including details of the tool(s) used, how many reviewers assessed each study and whether they worked independently, and if applicable, details of automation tools used in the process. | Page 7 |
| Effect measures | 12 | Specify for each outcome the effect measure(s) (e.g. risk ratio, mean difference) used in the synthesis or presentation of results. | Page 7 |
| Synthesis methods | 13a | Describe the processes used to decide which studies were eligible for each synthesis (e.g. tabulating the study intervention characteristics and comparing against the planned groups for each synthesis (item #5)). | Pages 5-7 |
|  | 13b | Describe any methods required to prepare the data for presentation or synthesis, such as handling of missing summary statistics, or data conversions. | Page 7 |
|  | 13c | Describe any methods used to tabulate or visually display results of individual studies and syntheses. | Page 7 |
|  | 13d | Describe any methods used to synthesize results and provide a rationale for the choice(s). If meta-analysis was performed, describe the model(s), method(s) to identify the presence and extent of statistical heterogeneity, and software package(s) used. | Page 7 |
|  | 13e | Describe any methods used to explore possible causes of heterogeneity among study results (e.g. subgroup analysis, meta-regression). | Page 7 |
|  | 13f | Describe any sensitivity analyses conducted to assess robustness of the synthesized results. | Page 7 |
| Reporting bias assessment | 14 | Describe any methods used to assess risk of bias due to missing results in a synthesis (arising from reporting biases). | Page 7 |
| Certainty assessment | 15 | Describe any methods used to assess certainty (or confidence) in the body of evidence for an outcome. | Page 7 |
| **RESULTS** | | |  |
| Study selection | 16a | Describe the results of the search and selection process, from the number of records identified in the search to the number of studies included in the review, ideally using a flow diagram. | Page 8, Figure 1 |
|  | 16b | Cite studies that might appear to meet the inclusion criteria, but which were excluded, and explain why they were excluded. | n/a |
| Study characteristics | 17 | Cite each included study and present its characteristics. | Table 1, Supplementary file 2, Pages 8-10 |
| Risk of bias in studies | 18 | Present assessments of risk of bias for each included study. | Page 10, Supplementary material 3 |
| Results of individual studies | 19 | For all outcomes, present, for each study: (a) summary statistics for each group (where appropriate) and (b) an effect estimate and its precision (e.g. confidence/credible interval), ideally using structured tables or plots. | Figure 2, Figure 3, Supplementary material 4 |
| Results of syntheses | 20a | For each synthesis, briefly summarise the characteristics and risk of bias among contributing studies. | Page 8-12 |
|  | 20b | Present results of all statistical syntheses conducted. If meta-analysis was done, present for each the summary estimate and its precision (e.g. confidence/credible interval) and measures of statistical heterogeneity. If comparing groups, describe the direction of the effect. | Page 11, page 13, Supplementary material 4 |
|  | 20c | Present results of all investigations of possible causes of heterogeneity among study results. | Page 12, Supplementary material 4 |
|  | 20d | Present results of all sensitivity analyses conducted to assess the robustness of the synthesized results. | Page 12, Supplementary material 4 |
| Reporting biases | 21 | Present assessments of risk of bias due to missing results (arising from reporting biases) for each synthesis assessed. | Page 10, Supplementary material 3 |
| Certainty of evidence | 22 | Present assessments of certainty (or confidence) in the body of evidence for each outcome assessed. | Page 11, page 13, Supplementary material 4 |
| **DISCUSSION** | | |  |
| Discussion | 23a | Provide a general interpretation of the results in the context of other evidence. | Page 14 |
|  | 23b | Discuss any limitations of the evidence included in the review. | Pages 15-16 |
|  | 23c | Discuss any limitations of the review processes used. | Pages 16-17 |
|  | 23d | Discuss implications of the results for practice, policy, and future research. | Pages 15-17 |
| **OTHER INFORMATION** | | |  |
| Registration and protocol | 24a | Provide registration information for the review, including register name and registration number, or state that the review was not registered. | Page 5 |
|  | 24b | Indicate where the review protocol can be accessed, or state that a protocol was not prepared. | Page 5 |
|  | 24c | Describe and explain any amendments to information provided at registration or in the protocol. | Page 5 |
| Support | 25 | Describe sources of financial or non-financial support for the review, and the role of the funders or sponsors in the review. | Page 17 |
| Competing interests | 26 | Declare any competing interests of review authors. | Page 17 |
| Availability of data, code and other materials | 27 | Report which of the following are publicly available and where they can be found: template data collection forms; data extracted from included studies; data used for all analyses; analytic code; any other materials used in the review. | Page 17 |
